# Multi-Stage Thermal Modelling of Extrusion-Based Polymer Additive Manufacturing

**DOI:** 10.3390/polym15040838

**Published:** 2023-02-08

**Authors:** Jiong Yang, Hexin Yue, Wajira Mirihanage, Paulo Bartolo

**Affiliations:** 1 Department of Mechanical, Aerospace and Civil Engineering, School of Engineering, University of Manchester, Manchester M13 9PL, UK; 2 Department of Materials, School of Natural Science, University of Manchester, Manchester M13 9PL, UK; 3 Singapore Centre for 3D Printing, School of Mechanical and Aerospace Engineering, Nanyang Technological University, 50 Nanyang Avenue, Singapore 639798, Singapore

**Keywords:** 3D printing, CFD, extrusion, process modelling

## Abstract

Additive manufacturing is one the most promising fabrication strategies for the fabrication of bone tissue scaffolds using biodegradable semi-crystalline polymers. During the fabrication process, polymeric material in a molten state is deposited in a platform and starts to solidify while cooling down. The build-up of consecutive layers reheats the previously deposited material, introducing a complex thermal cycle with impacts on the overall properties of printed scaffolds. Therefore, the accurate prediction of these thermal cycles is significantly important to properly design the additively manufactured polymer scaffolds and the bonding between the layers. This paper presents a novel multi-stage numerical model, integrating a 2D representation of the dynamic deposition process and a 3D thermal evolution model to simulate the fabrication process. Numerical simulations show how the deposition velocity controls the spatial dimensions of the individual deposition layers and the cooling process when consecutive layers are deposited during polymer printing. Moreover, numerical results show a good agreement with experimental results.

## 1. Introduction

In an extrusion-based polymer additive manufacturing system, a thermoplastic material, in the form of a filament or pellets, is heated above the melting point inside the extrusion chamber and extruded out of the nozzle by applying pressure, mechanical forces or using a screw [1,2,3]. In the field of biomanufacturing, extrusion-based processes are extensively used for the fabrication of biodegradable and biocompatible 3D porous tissue engineering scaffolds or cell-laden constructs [4]. A wide range of materials including synthetic polymers, usually presenting high melting points, natural polymers with low processing temperatures, polymer-based composites and bioinks (hydrogels containing cells and growth factors) can be processed by extrusion-based additive manufacturing [5,6,7,8].

In the case of scaffold-based approaches, the most common in tissue engineering, extrusion-based additive manufacturing involves the solidification of polymer melts, as most polymers used in biomanufacturing are semi-crystalline polymers. Generally, the solidification process conditions determine a range of properties and influence the geometrical characteristics, mechanical properties, degradation kinetics and biological characteristics of the additive manufactured scaffolds [9]. During the polymer extrusion additive manufacturing process, materials are melted and then printed (deposited) layer by layer into a platform where they are cooled down. The melting process creates an amorphous semi-liquid viscous material. The material is then pushed through the nozzle into the printed platform. During this process, polymer chains tend to be aligned according to the printing direction [10]. The cooling process, strongly dependent on the thermal gradients, determines the crystallisation mechanism (e.g., crystal size, crystal orientation and crystallinity level), which influences the mechanical properties and degradation kinetics of printed scaffolds [11]. Therefore, it is critical to predict and control thermal transitions during the printing and cooling stages.

Several numerical models have been developed to predict temperature profiles during the 3D printing process [12,13,14]. However, these models are too simplistic and are based on ideal conditions; consequently, the results are less accurate. Compton and co-workers [15] developed a simulation tool for the evaluation of temperature distribution on multi-layer large-scale polymer composite structures. The authors used a thermal camera to record temperature changes during the printing process. Results showed that the model was not able to accurately predict temperature differences between layers. Moreover, the simulation tool was not able to provide sufficient detail on the temperature profiles, compromising potential microstructure analysis. Other researchers [16,17] used coarse mesh models to save computational time or adaptive mesh approaches based on ideal conditions that compromised the accuracy of the predicted results. Thermal transient finite element analysis (FEA) was also used by Brenken et al. [18] to simulate the thermal history of 3D printed acrylonitrile butadiene styrene (ABS) filaments. However, the authors were not able to simulate the thermal transitions during the printing process, critical for the evaluation of morphological changes, but only the cooling process after printing. As observed, previously reported studies were not able to simulate temperature changes and cooling processes during the printing process with free cooling in open atmosphere, and mostly considered low cooling rate conditions. Moreover, several studies also considered the cooling process after the printing process, which does not allow an understanding of the temperature changes prior to the fabrication of the overall part.

This paper proposes a new computational fluid dynamics (CFD) modelling approach to predict the overall temperature evolution in extrusion-based additive manufacturing. The model simulates the entire fabrication process, from material being extruded out of the nozzle, printed on the platform and cooled down to room temperature. A two-dimensional (2D) model with a fine mesh size and dynamic mesh controlling approach are used to simulate the filament extrusion and printing process. This 2D model significantly saves computational time, while the use of a dynamic mesh allows researchers to simulate the printing process in a more realistic way. Moreover, a three-dimensional (3D) model including filament, plate support and free air space is used to simulate the extruded filament cooling process. This 3D model allows researchers to obtain detailed information on the temperature distribution at different positions within the filament, allowing the determination of temperature gradients and cooling rates during the printing process and after fabrication.

## 2. Model Development

### 2.1. Numerical Model

Physically, the rate of mass change of a fluid is equal to the rate of fluid flow into a controlled volume. According to the conservation law, the general continuity equation is written as follows [19]:

(1) $$\frac{\partial \rho }{\partial t}+\nabla \cdot j=\sigma$$

where ∇ is the divergence; ρ is the density; j is the flux of the material, defined as j=ρv with v being the velocity of the respective direction; t is the time; and σ is the generation of fluid mass change. As σ is usually equal to zero when an incompressible liquid is considered, Equation (1) for a given phase *i* can be rewritten as:

(2) $$\frac{\partial \rho_{i}}{\partial t}+\nabla \cdot \left(\rho_{i}v\right)=0$$

According to the conservation of momentum, any change of momentum on a fluid inside a controlled volume is equal to the momentum in the fluid and the action of the external forces acting on the fluid. Therefore, the momentum conservation equation is given by [20]:

(3) $$\frac{\partial }{\partial t}\left(\rho_{i}\vec{v}\right)+\nabla \cdot \left(\rho_{i}\vec{v}\vec{v}\right)=-\nabla p+\nabla \cdot \left(\overset{\equiv }{\tau }\right)+\rho_{i}\vec{g}+\vec{F}$$

where p is the static pressure, $\overset{\equiv }{\tau }$ is the stress tensor, and $\rho \vec{g}$ and $\vec{F}$ are the gravitational body forces and external body forces, respectively.

Moreover, as the total energy in a closed system remains constant, the energy conservation equation for an incompressible flow with constant density is given by [20]:

(4) $$\rho_{i}c_{p}\left[\frac{\partial T}{\partial t}+\left(\vec{V}\cdot \nabla T\right)\right]=k\nabla^{2}T+S_{h}$$

where $c_{p}$ is the specific heat, k is the thermal conductivity of the material, T represents the temperature and $S_{h}$ is the viscous dissipation function, which governs the rate of heat transformation associated with the solid to fluid transformation.

**Figure 1:**
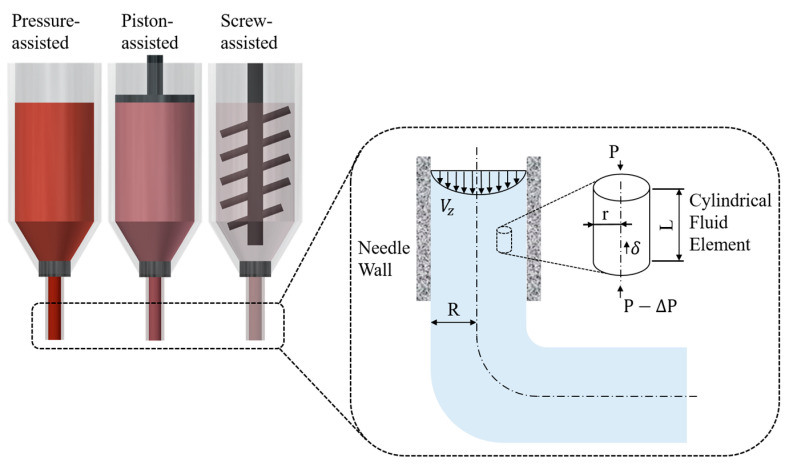
Schematic representation of the extrusion-based fluid flow on a small pipe (the drawn velocity profile is for an ideal material).

To simulate the material flow in a cylindrical needle (Figure 1), the following assumptions were considered:

1. The air compressibility can be ignored;
2. The fluid flow is incompressible and time-independent;
3. The material inside the needle exhibits a laminar flow and there is no slip between the material and needle wall;
4. The fluid exhibits a non-Newtonian flow behaviour and ideal thermal conditions.

Suitable materials for extrusion-based additive manufacturing must exhibit a shear-thinning behaviour, characterised by a decrease in the viscosity by increasing the shear rates [21]. In addition, the material thermal properties and parameters may slightly fluctuate during the cooling stage. However, in the simulations, the thermal boundaries (external environment of the model) and parameters remain constant and are assumed as ideal thermal conditions. Based on these assumptions, material state in a cylindrical needle can be described based on the linear momentum balance of a fluid element with radius r and length L (Figure 1):

(5) $$P\left(\pi r^{2}\right)=\left(P-\Delta P\right)\pi r^{2}+2\pi rL\tau_{rz}$$

where P is the applied pressure, ΔP is the pressure drop, with the absolute value being considered for simulation purposes, and $\tau_{rz}$ represents the shear stresses on the cylindrical element inside the needle.

Rearranging Equation (5) we obtain:

(6) $$\tau_{rz}=\left(\frac{r}{2}\right)\left(\frac{\Delta P}{L}\right)$$

The shear rate $\dot{\gamma }$ inside the needle describes the rate of velocity change in the radial direction:

(7) $$\dot{\gamma }=\frac{dv_{z}}{dr}$$

For a non-Newtonian material, the shear stress generated during the printing process can be represented as a function of the shear rate according to the following equation [22]:

(8) $$\tau_{rz}=\mu \left(\frac{dv_{z}}{dr}\right)=K\cdot {\dot{\gamma }}^{n}$$

where K is the consistency flow index, μ is the viscosity of a non-Newtonian fluid and n represents the flow behaviour index.

By combining Equations (6)–(8), the velocity along the needle length can be obtained after integration:

(9) vz=∫rRγ˙dr  →  vz=nn+1ΔPR2KL1nR1−rRn+1n

where R is the needle radius. As shown in Figure 1, the velocity profile within the needle in the radial direction is not uniform, reaching a maximum at the centre of the needle and being zero at the needle wall. Therefore, the flow rate Q can be calculated by integrating the velocity over the cross section:

(10) Q=∫0R2πrvzdr  →  Q=πR3ΔPR2KL1nn3n+1

For a given Q, the extruded volume $V_{s}$ within a period of time t is given by:

(11) $$V_{s}=Q\cdot t$$

Assuming that the extruded volume forms a cylindrical strand with uniform diameter d and length $L_{d}$, the extruded volume can be expressed as follows:

(12) $$V_{s}=\pi {\left(\frac{d}{2}\right)}^{2}L_{d}$$

Moreover, considering $v_{p}$ the nozzle horizontal movement velocity, the printed filament length can be calculated as follows:

(13) $$L_{d}=v_{p}\cdot t$$

Combining Equations (11)–(13), it is possible to obtain the following equation:

(14) $$v_{p}=\frac{4Q}{\pi d^{2}}$$

that allows researchers to determine the filament diameter as follows:

(15) $$d=\sqrt{\frac{4Q}{\pi v_{p}}}$$

### 2.2. Multi-Stage Approach

The ANSYS Workbench software (ANSYS, United States) was used as the solver platform for the CFD simulation. The numerical simulations were carried out using multi-phases model, energy and thermal equations, considering an incompressible and non-Newtonian material. These models were used to simulate both temperature history and dimensional characteristics of filaments during the extrusion process.

**Figure 2:**
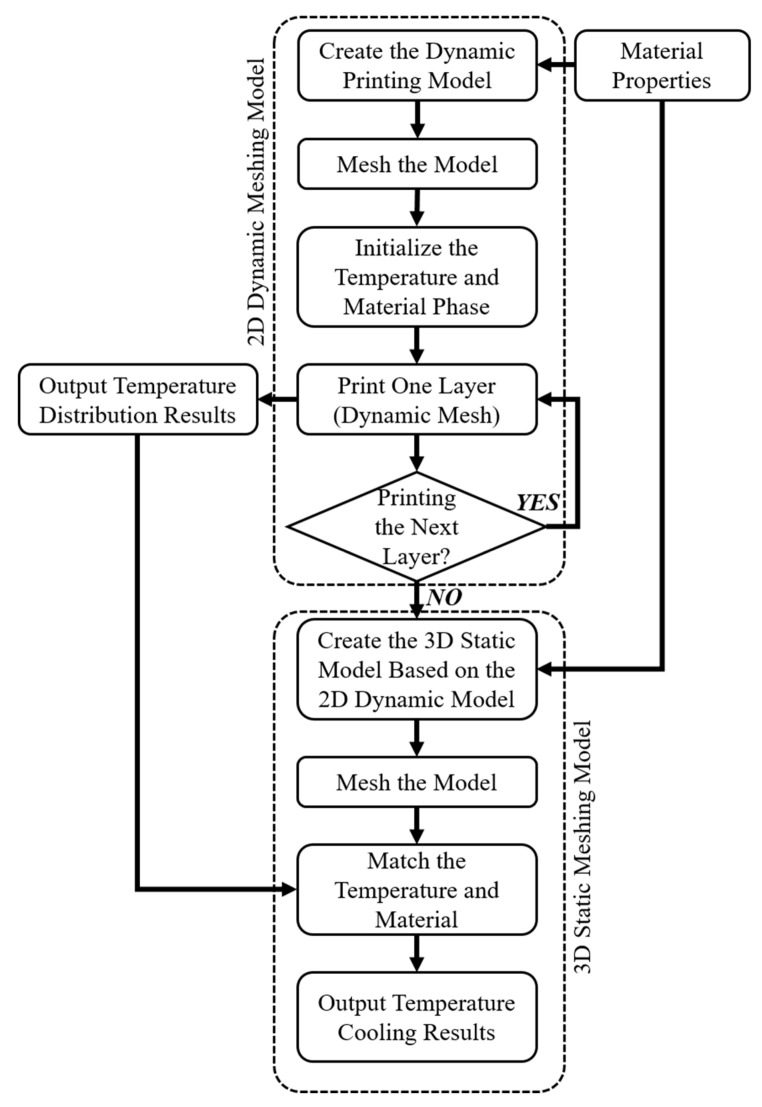
Flowchart describing the multi-stage thermal simulation model to estimate the multiple layer filament temperature history.

In this study, a new multi-stage thermal simulation model was developed by combining a 2D dynamic meshing model and a 3D static meshing model to predict the extrusion-based polymer thermal evolution during the extrusion and printing process. Figure 2 shows the general simulation procedure using these two models. The 2D model was implemented at the first stage for the material extrusion simulation, while the 3D model was used for the post-extrusion cooling stage. The 2D model was used to simulate the material being printed out of the nozzle and deposited on the platform in a computationally efficient way. Once the printing process was completed, the temperature distribution results were exported from the 2D model into the 3D model and used to simulate the final stage of the cooling process. The temperature profile was loaded as a field function in the symmetrical plane (y-z plane in the 3D model). Since the filament has a large length–width ratio, the temperature was considered to be symmetrically distributed in the x direction. The combined use of these two models allows researchers to save computational time without the need to oversimplify the numerical models, allowing them to obtain more realistic results. Simulations were conducted considering both one-layer and two-layer filament cases.

### 2.3. 2D Dynamic Meshing Model

**Figure 3:**
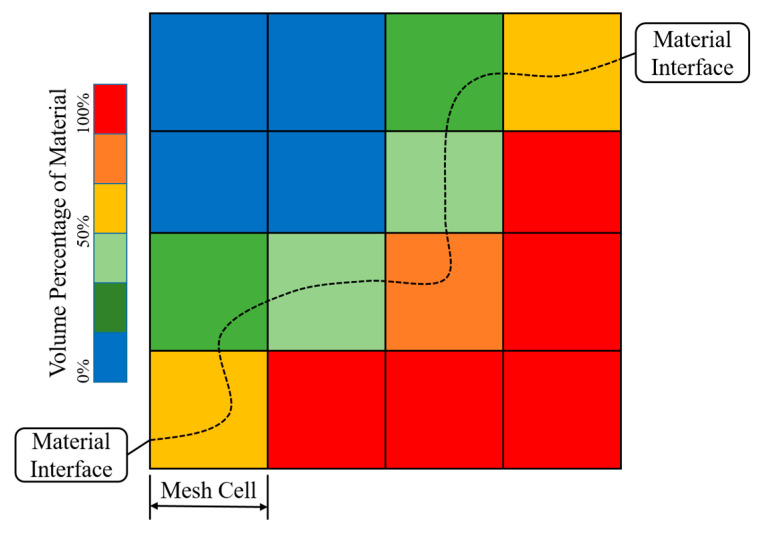
Schematic representation of the VOF method in the ANSYS multi-phase model.

The 2D dynamic meshing model was considered to predict the polymer shape and temperature distribution during the extrusion printing process. In this case, the volume of fluid (VOF) method was used to track the interface between the melted polymer and air. Figure 3 represents the liquid volume fraction β in each computational cell volume (in this case 2D). Each cell assumes a red colour when β is equal to one and a blue colour when β is equal to zero. Interface cells (0 < β < 1) assume a colour gradient between red and blue.

**Figure 4:**
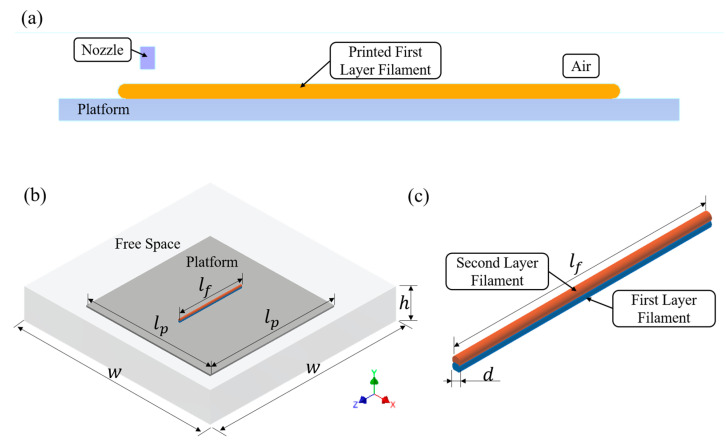
Computational domain and boundary conditions of (**a**) 2D dynamic meshing model; (**b**) isometric view of the 3D thermal evolution model; and (**c**) zoomed view of the two-layer filament.

The dynamic meshing is controlled by the horizontal velocity of the nozzle and the material extrusion out of the nozzle towards the printing platform. The movement of the nozzle and printing platform are controlled by user-assigned parameters to simulate the process (motion file). Therefore, the dynamic meshing model can critically simulate the polymer flows where the shape of the domain changes with time due to motion on the domain boundaries. In the simulation cases, the nozzle assumed a constant velocity at the horizontal direction. The first and second layers were printed in an opposite direction by attributing a positive and negative velocity to the nozzle. Figure 4a shows an example of a 2D model with an extrusion nozzle, a platform (aluminium) and free space (air). Materials are extruded out from the nozzle at an extrusion velocity $v_{e}$ and printed at a printing velocity $v_{p}$.

### 2.4. 3D Static Meshing Model

The extruded filaments were placed on a platform. However, the dimensions of the printed filaments and overall scaffolds were usually relatively small compared with the dimensions of the printing platform. Therefore, our model considers a relatively large aluminium platform surrounded by a large atmosphere space. Although this design requires more computer capacity and computational time, it significantly improves the quality of the simulations, which become more realistic and precise.

Figure 4b shows the 3D model and boundary conditions considered for the cooling simulation stage. The initial temperature distribution and filament dimensions were imported from the 2D model. In all considered cases the filament length, $l_{f},$ was equal to 10 mm, while the filament diameter d varied as a function of both $v_{e}$ and $v_{p}$. The 3D printing process was performed on a square aluminium platform with dimensions $l_{p},$ w and h, considering filaments with diameter d as shown in Figure 4b,c.

## 3. Materials and Implementation

Polycaprolactone (PCL) in a pellet form supplied by PerstorpCaprolactones (Warrington, UK) was the biomaterial considered for the simulations. This is a synthetic semi-crystalline polyester polymer commonly used for the fabrication of bone tissue engineering scaffolds [23,24], presenting a melting temperature of $336.58\;K\;\left(63.43\;{}^{\circ}C\right)$, density of $1145\;\mathrm{kg}/m^{3}$, specific heat of $1450\;j/\left(\mathrm{kg}\cdot K\right)$ and thermal conductivity of $0.14\;w/\left(m\cdot K\right).$ A non-Newtonian rheological model was considered to simulate the PCL printing process assuming the following parameters: power law index (n) of 0.39; activation energy of 33.9 kJ/mol [25]; and consistency index of $5.3\;Pa\cdot S^{n}$ [26]. Additionally, Table 1 presents the key thermal material properties considered for the printing platform (aluminium) and free space (air). The dimensions of the aluminium platform were $l_{p}=20\;\mathrm{mm},$ w=30 mm and h=5 mm.

The simulations were performed using high performance computing (HPC) cluster (43 nodes of 2 × 16-core Intel Xeon Gold 6130 CPU @ 2.10GHz + 192GB RAM + 100Gb/s (4X EDR) mlx5 Mellanox InfiniBand). Triangle elements with 50 μm of size were used in the 2D dynamic meshing model, while for the 3D static meshing model tetrahedron elements were used with 50 μm of size for the filament and 500 μm of size for both platform and atmosphere area.

**Table 1:** The thermal properties of the platform material (aluminium) [27] and air [28].

**Property**	**Air**	**Aluminium**
Density (kg/m^3^)	1.225	2719
Specific Heat (j/kg·k)	1006.43	871
Thermal Conductivity (w/m·k)	0.0242	202.4
Viscosity (kg/m∙s)	1.7894e−05	/
Heat Transfer Coefficient (w/(m^2^∙K))	10	60

## 4. Experimental Work for Numerical Validation

**Figure 5:**
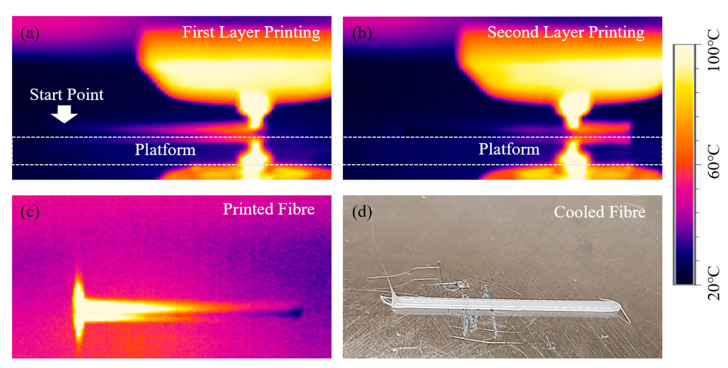
Measured temperature distribution on a two-layer filament model at (**a**) the first layer during the printing process; (**b**) the second layer during the printing process; (**c**) the printed fibre; (**d**) the cooled fibre. Printing parameters: extrusion velocity of 3.5 mm/s and printing velocity of 3.5 mm/s.

The simulation scenarios were experimentally extruded using a 3D-BioPlotter (Envision tech, Gladbeck, Germany) by printing two-layer filaments, considering different extrusion velocities (2.4–3.5 mm/s) and printing velocities (2.5–3.5 mm/s). The extrusion velocities were controlled by adjusting the pressure values (4–6 bar). The actual temperature readings were obtained through thermal imaging using a thermalIMAGER TIM 160S (Micro Epsilon, Wirral, UK). This camera enables up to 1 kHz for fast process, allowing the detection of temperature distributions at each time step with good precision [29]. The thermal imaging camera was placed directly in front of the printer to detect the temperature history on the polymer surface during printing. Surface temperature images were captured at each second. An example of measured temperature fields during the printing process is presented in Figure 5. Centre points on the first and second printed layers were considered to determine temperature changes during both the printing and cooling phases. The temperature of the printed platform was kept at 15 °C during the printing and cooling stages. Experiments were performed in duplicate (no significant differences between experiments) and the results are presented as average values.

## 5. Results and Discussion

### 5.1. Filament Morphology

**Figure 6:**
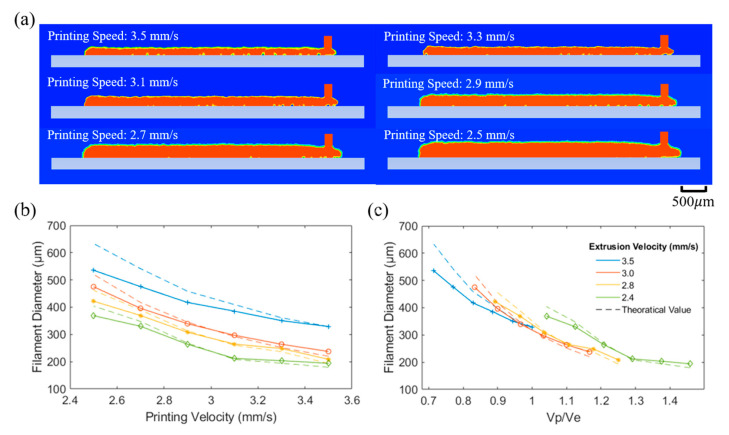
(**a**) Filament morphology considering the dynamic meshing model, (**b**) filament diameter versus printing velocity and (**c**) filament diameter versus $v_{p}/v_{e}$.

The numerical predictions of the filament shape were obtained using the 2D multi-phases model with dynamic mesh motion. Results presented in Figure 6 show that the filament diameter remains almost constant during the printing process. For higher printing speeds it is possible to observe (Figure 6a) the presence of some bubbles between the filament domain and the platform. Figure 6b also shows that the filament diameter increases by decreasing $v_{p}$ from 3.5 mm/s to 2.5 mm/s, and $v_{e}$ from 3.5 mm/s to 2.4 mm/s. As observed from the simulation results, it is possible to generate a fine filament shape when the $v_{e}$ and $v_{p}$ are controlled in a specific matching range. Moreover, results show that the filament shape can be predicted with a high level of accuracy regarding the theoretical values. Figure 6c presents the results based on the ratio between the printing velocity and the extrusion velocity,$\;v_{p}/v_{e}$. As observed, the filament diameter decreases by increasing $v_{p}/v_{e}$, which is aligned with previously reported results [30]. Moreover, for the same $v_{p}/v_{e}$ values, filaments printed with different extrusion velocities present similar filament diameters, which is in accordance with the theoretical models.

### 5.2. First Layer Temperature Evolution

**Figure 7:**
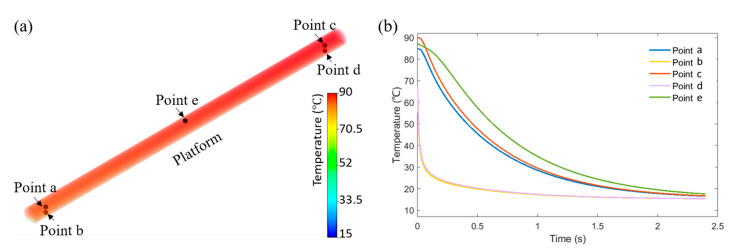
(**a**) Initial temperature distribution and (**b**) area-based cooling curves at different positions.

Figure 7a presents the temperature distribution in the 3D printed filament at time 0 s. The cooling curves considering different locations (points a to e) in the filament are presented in Figure 7b. As observed, positions close to the printing platform (points b and d) present an extremely fast cooling rate. However, point a and point c exhibit a similar temperature profile, excepting the initial temperature due to the printing time delay. As observed, at higher positions at the centre of the filament (point e), the cooling rate is lower with reduced heat flux between the interface polymer–air (top surface of the filament) and polymer–aluminium (bottom surface of the filament). Results also show that the thermal evolution model can clearly describe the cooling process of the polymer after extrusion.

**Figure 8:**
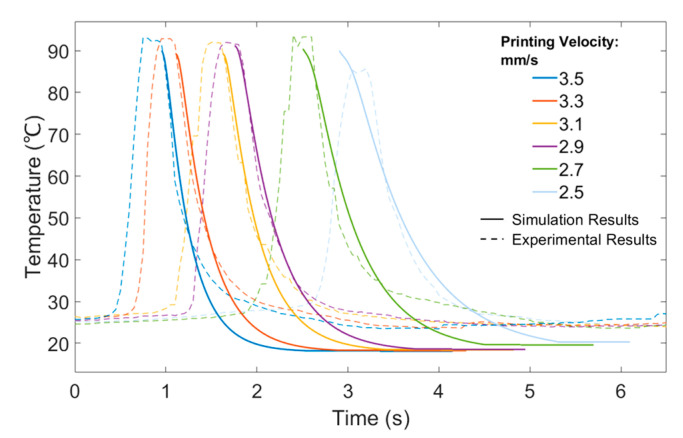
Comparison between numerical and experimental thermal imaging results of the temperature history for the one-layer filament case.

Several simulation cases considering different printing velocities were also investigated and the numerical results were experimentally validated through thermal imaging. Results are presented in Figure 8 and show a high level of accuracy between numerical and experimental data. The polymer was extruded at 100 °C and printed on the platform. A fan was used to keep the temperature at the printing area as constant as possible during the experimental procedure (around 20 °C). As the printing velocity decreased, it took more time for the printed filaments to cool down and fully solidify. These results confirm the suitability of the proposed models to describe the printing process.

**Figure 9:**
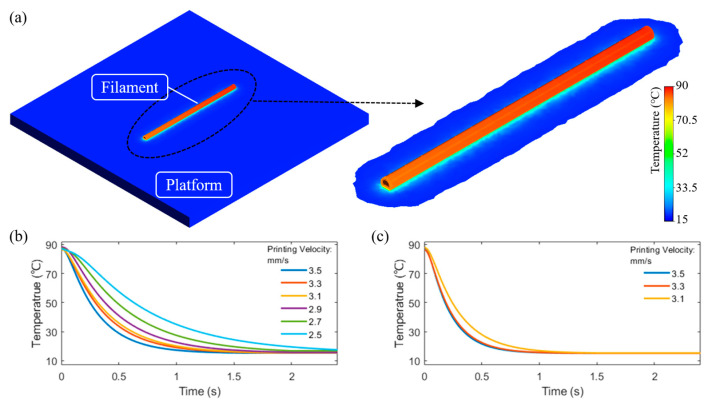

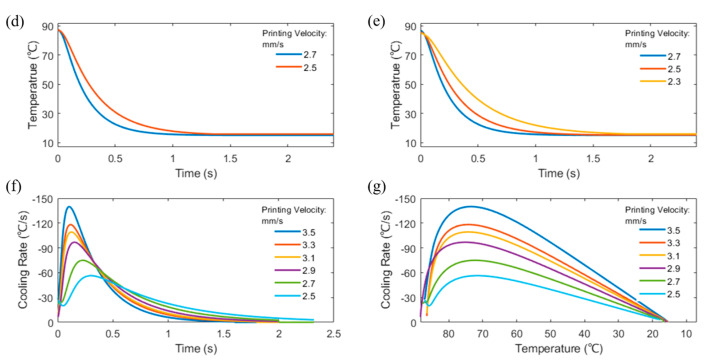
(**a**) Example of temperature distribution on a printed filament at time 0 s; temperature decreasing results as a function of printing velocity for (**b**) 3.5 mm/s of extrusion velocity; (**c**) 3.0 mm/s of extrusion velocity; (**d**) 2.8 mm/s of extrusion velocity and (**e**) 2.4 mm/s of extrusion velocity; (**f**) filament cooling rate versus time and (**g**) filament cooling rate versus temperature for an extrusion velocity of 3.5 mm/s and different printing velocities.

**Figure 10:**
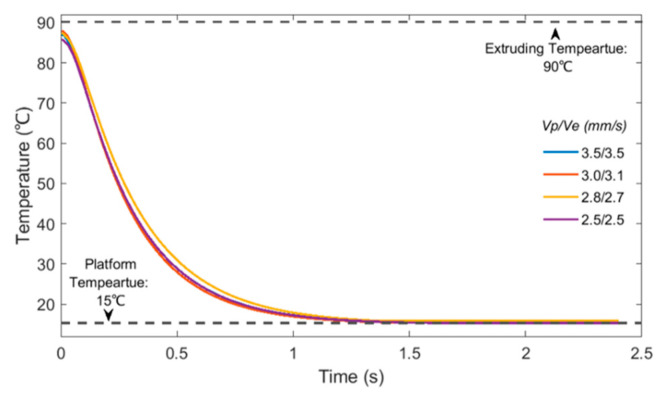
Temperature cooling profiles for different values of $v_{p}/v_{e}$.

Figure 9a shows the temperature distribution on a printed filament at time 0 s. The effect of both printing and extrusion velocity is presented in Figure 9b–e. As observed, the temperature curve rapidly drops after extrusion, and then the temperature decrease rate gradually slows down. Figure 9f,g describe the variation of the cooling rate as a function of time and temperature, respectively, for an extrusion velocity of 3.5 mm/s. Results show that the maximum cooling rate decreases by decreasing the printing velocity and the cooling rate peak shifts to higher cooling times by decreasing the printing speed. Moreover, the maximum cooling rate occurs for a temperature around 72 °C (345 K) for all considered printing velocities. Similar results were obtained for the other extrusion velocities (Figure S1, Supplementary Information). The cooling process takes more time by decreasing the printing velocity, which can be related to the volume of printed material reflected on the larger filament diameters obtained at low printing velocities. Figure 10 shows the temperature decrease as a function of time for filaments presenting similar diameters and considering different $v_{p}/v_{e}$ ratios. As observed, all cases present similar cooling profiles. These results showed that the ratio between the printing velocity and the extrusion velocity is a critical parameter to control the filament diameter and the cooling process.

### 5.3. Temperature Evolution on the Two-Filament Model

**Figure 11:**
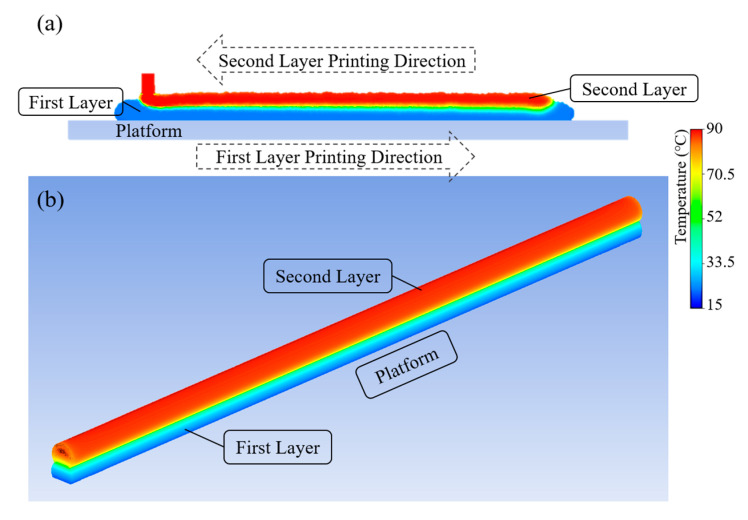
Temperature distribution on the two-layer filament model at the end of the printing process considering (**a**) the 2D dynamic meshing model and (**b**) the 3D static meshing model.

To investigate the heat transfer between the molten filament and the previously printed and cooled filament, the second layer was assumed to be printed directly on top of the first layer with a backward printing direction. Results for both 2D and 3D models are presented in Figure 11. After a certain time, which depends on the printing velocity, the first layer cools down and solidifies. Once the second layer is printed, the first layer is re-heated, inducing a re-crystallisation process and contributing to the adhesion between the two filaments.

**Figure 12:**
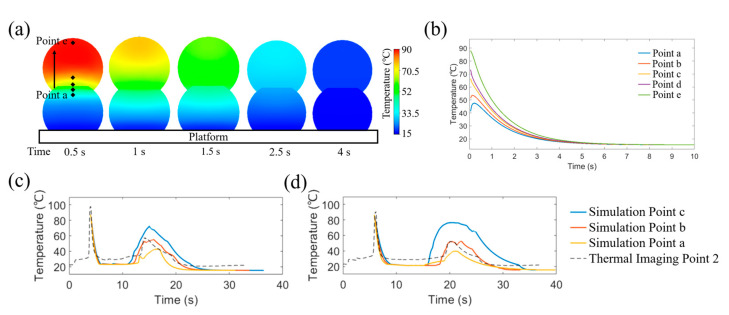
Temperature results of a two-layer filament model with $v_{e}=3.5\;\mathrm{mm}/s$ and $v_{p}=3.5\;\mathrm{mm}/s$ in (**a**) section view of the filament temperature distribution at different times and (**b**) relative temperature cooling profile at different positions (time was measured from the end of the deposition of the second layer). Vertical temperature variations and thermal imaging of cases with (**c**) $v_{e}/v_{p}=3.5/3.5$ mm/s and (**d**) $v_{e}/v_{p}=3.5/2.7$ mm/s (time was corrected to the corresponding experimental time).

Figure 12a shows the temperature distribution at the cross-section of two consecutive printed filaments at different time points. After printing the second layer on top of the first one (0 s), it is possible to observe a significant temperature gradient. The first layer filament has a relatively low temperature near the platform, almost close to room temperature, while the top area in contact with the second filament is re-heated to a higher temperature. Moreover, the bottom of the second filament, which is in contact with the first layer, shows lower temperatures than the top regions. The temperature gradient decreases with time and after 4 s both filaments are at a similar temperature. The cooling profiles at different positions (the five points shown in Figure 12a, positions from platform: point a 200 µm, point b 230 µm, point c 250 µm, point d 300 µm and point e 550 µm) as a function of time are presented in Figure 12b. As expected, the temperature of the first layer rises for a short period of time, between 0 s and 0.5 s, because of the heated second layer, and then starts to decrease. Full-process temperature history is presented in Figure 12c,d, including reference results obtained by thermal imaging. As observed, point **c** (at the interface of the two layer filaments) has the highest peaking point on the second heating progress, presenting a temperature around 80 °C. Point **b** (20 µm to the interface) and point **a** (50 µm to the interface) show a similar heating process. However, due to the relatively larger distance to the second printed filament, the peaking points decrease with less heat transfer from the upper filament. Therefore, the peak temperature of the second heating process is dramatically dependent on the distance to the second layer filament, inducing a dynamic temperature gradient evolution on those points. However, due to the range of printed filament diameters (between 200 μm and 500 μm in both numerical and experimental cases) and the precision of the thermal camera, it was only possible to record one reference point in the vertical direction, making difficult to analyse the temperature gradient influence. Therefore, the temperature gradient aspects predicted by the proposed model were only partially confirmed.

The proposed multi-stage model not only provides accurate results but is also computationally less expensive. Alone, the dynamic mesh, which resets the mesh at each time step of the domain motion, requires relatively demanding computational resources. In our case, a 50 μs time-step takes around 4 h per filament to compute. However, the 2D model, considering a dynamic meshing that allows the trajectory of the moving nozzle to be determined by uploading a motion file, reduces the computational time and memory space and simulates the filament morphology at each step during the fabrication process. The static mesh, employed for the 3D model, handles only diffusive heat transport, and greatly reduces computational time (around 2 h for the entire cooling process) without oversetting the mesh at each time step.

## 6. Conclusions

A novel multi-stage thermal simulation model, combining both 2D dynamic meshing and 3D static meshing models, was developed to predict the temperature distribution and history of multiple layer filaments during polymer extrusion. The model was used to simulate the material extrusion out of the nozzle and the printing process of PCL, and the results were experimentally validated. Even though the exchanging temperature data between the two models introduces a moderately high memory requirement for the implementation, the combination of a 2D dynamic meshing model and a 3D static meshing model allows researchers to obtain results with a reasonably good accuracy while limiting the computational resource requirements to a moderate level.

The simulation model was used to investigate the material thermal evolution and temperature distribution of polymer filaments during the extrusion, printing and cooling steps in an extrusion-based additive manufacturing. The effects of extrusion and printing velocity on the temperature profiles were investigated and the results compared with experimental ones obtained through thermal imaging. The boundary conditions of the numerical model were designed based on the experimental work, considering PCL as the testing material. The results show that the increase in both extrusion and printing velocity accelerates the cooling process, which also has an impact on the filament diameter that decreases by decreasing the printing velocity and increases by increasing the extrusion velocity. Moreover, the simulation model allows researchers to obtain temperature profiles at the interface between two printed filaments, which enables them to control filament bonding. Results show a reasonable agreement between numerical and experimental results.

The multi-phase thermal estimation model can be used to simulate the thermal behaviour of different materials including different types of polymeric and polymer-based composite material. The accurate temperature distribution obtained by the proposed model potentially allows for the investigation of the morphological development process during the melting and cooling steps, enabling researchers to determine the evolution of crystallinity and re-crystallization mechanisms during the fabrication process that can ultimately be linked with the mechanical characteristics of the printed filaments. Therefore, the multi-phase numerical model can be considered as a platform and a method for other investigators to explore the polymer thermal evolutions during extrusion-based additive manufacturing.

## Data Availability

Not applicable.

## Supplementary Materials

The following supporting information can be downloaded at: https://www.mdpi.com/article/10.3390/polym15040838/s1, Figure S1: Filament cooling rate results.
