# Supplementary material for: Multi-Stage Thermal Modelling of Extrusion-Based Polymer Additive Manufacturing
Source: Polymers (Basel). 2023 Feb 8;15(4):838. doi: 10.3390/polym15040838 (PMC9966975; doi:10.3390/polym15040838)
Supplement: Supplementary file 1 [file polymers-15-00838-s001.zip › polymers-2089220-supplementary.pdf]

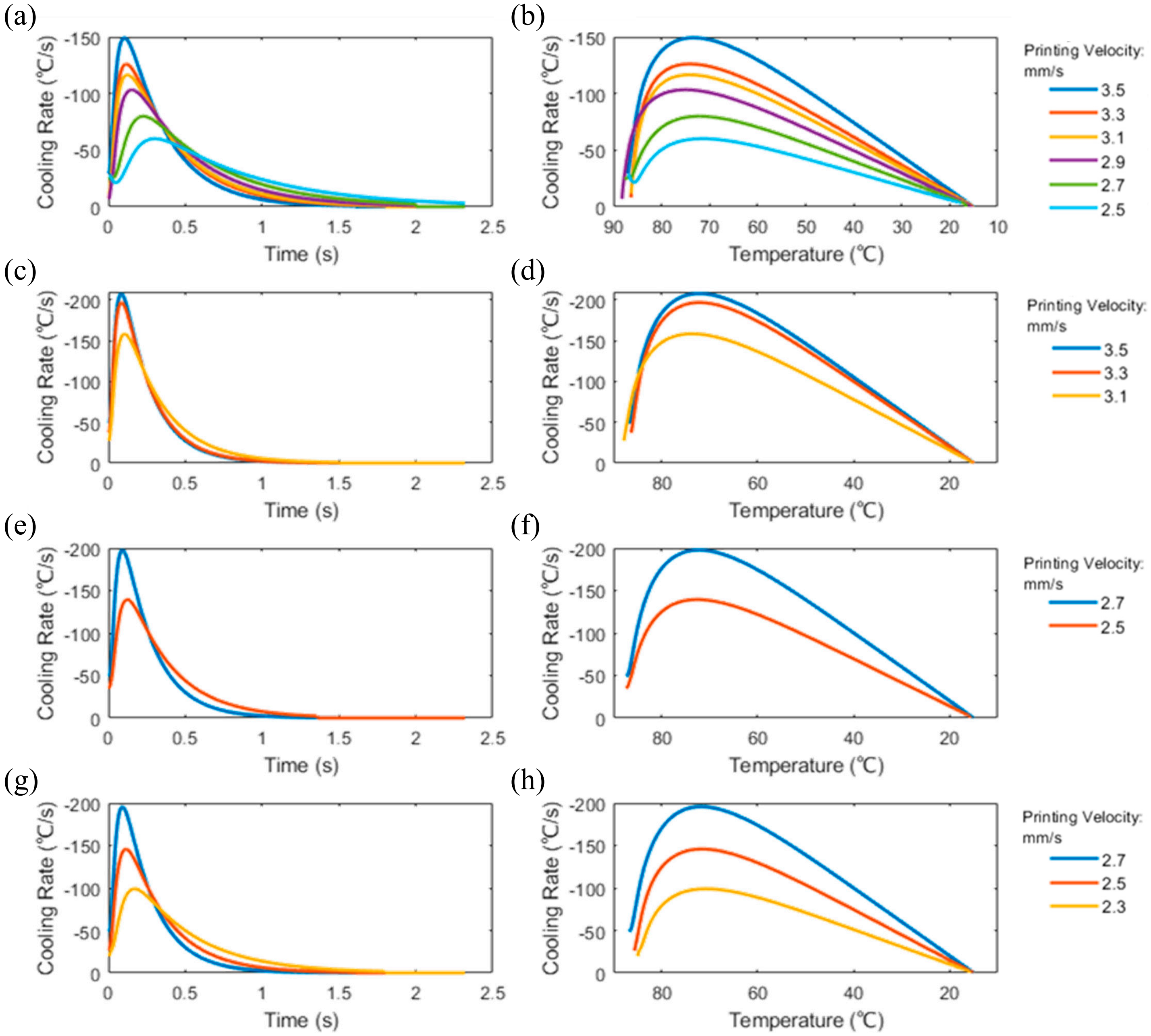

Figure S1. Filament cooling rate results of (**a**) cooling rate versus time with extrusion velocity of 3.5 mm/s; (**b**) cooling rate versus temperature with extrusion velocity of 3.5 mm/s; (**c**) cooling rate versus time with extrusion velocity of 3.0 mm/s; (**d**) cooling rate versus temperature with extrusion velocity of 3.0 mm/s; (**e**) cooling rate versus time with extrusion velocity of 2.8 mm/s; (**f**) cooling rate versus temperature with extrusion velocity of 2.8 mm/s; (**g**) cooling rate versus time with extrusion velocity of 2.4 mm/s; (**h**) cooling rate versus temperature with extrusion velocity of 2.4 mm/s.
